# Glycoform Modification of Secreted Recombinant Glycoproteins through Kifunensine Addition during Transient Vacuum Agroinfiltration

**DOI:** 10.3390/ijms19030890

**Published:** 2018-03-17

**Authors:** Yongao Xiong, Qiongyu Li, Muchena J. Kailemia, Carlito B. Lebrilla, Somen Nandi, Karen A. McDonald

**Affiliations:** 1 Department of Chemical Engineering and Materials Science, University of California, Davis, CA 95616, USA; yxiong@ucdavis.edu (Y.X.); snandi@ucdavis.edu (S.N.); 2 Department of Chemistry, University of California, Davis, CA 95616, USA; qyuli@ucdavis.edu (Q.L.); jkmuchena@ucdavis.edu (M.J.K.); cblebrilla@ucdavis.edu (C.B.L.); 3 Department of Biochemistry and Molecular Medicine, University of California, Davis, CA 95616, USA; 4 Global HealthShare, Molecular and Cellular Biology, University of California, Davis, CA 95616, USA

**Keywords:** *N*-glycosylation, kifunensine, *Nicotiana benthamiana*, transient protein expression, Fc-fusion protein, apoplast wash fluid

## Abstract

Kifunensine, a potent and selective inhibitor of class I α-mannosidases, prevents α-mannosidases I from trimming mannose residues on glycoproteins, thus resulting in oligomannose-type glycans. We report for the first time that through one-time vacuum infiltration of kifunensine in plant tissue, N-linked glycosylation of a recombinant protein transiently produced in whole-plants shifted completely from complex-type to oligomannose-type. Fc-fused capillary morphogenesis protein 2 (CMG2-Fc) containing one *N*-glycosylation site on the Fc domain, produced in *Nicotiana benthamiana* whole plants, served as a model protein. The CMG2-Fc fusion protein was produced transiently through vacuum agroinfiltration, with and without kifunensine at a concentration of 5.4 µM in the agroinfiltration suspension. The CMG2-Fc *N*-glycan profile was determined using LC-MS/MS with a targeted dynamic multiple reaction monitoring (MRM) method. The CMG2-Fc expression level in the infiltrated plant tissue and the percentage of oligomannose-type *N*-glycans for kifunensine treated plants was 874 mg/kg leaf fresh weight (FW) and 98.2%, respectively, compared to 717 mg/kg leaf FW and 2.3% for untreated plants. Oligomannose glycans are amenable to in vitro enzymatic modification to produce more human-like *N*-glycan structures that are preferred for the production of HIV-1 viral vaccine and certain monoclonal antibodies. This method allows glycan modifications using a bioprocessing approach without compromising protein yield or modification of the primary sequence, and could be expanded to other small molecule inhibitors of glycan-processing enzymes. For recombinant protein targeted for secretion, kifunensine treatment allows collection of glycoform-modified target protein from apoplast wash fluid (AWF) with minimal plant-specific complex *N*-glycan at higher starting purity and concentration than in whole-leaf extract, thus simplifying the downstream processing.

## 1. Introduction

Plant-based pharmaceutical production is appealing given its inexpensive facility and production cost, linear scale-up, the absence of animal pathogens, and capability to produce complex proteins and perform post-translational modifications, which overcomes one or more drawbacks of traditional recombinant protein expression systems such as animal cell culture and bacterial fermentation [1,2]. Much work has been carried out using stably transformed plants, but the significantly reduced development and production timeline makes transient expression of proteins in whole plants a particularly attractive option, cutting the time to bring critical medications to the market during a pandemic [2]. Vacuum agroinfiltration is the most widely used method for uniformly introducing agrobacterium harboring an expression cassette containing a gene of interest into plant tissue given its natural ability to transfer T-DNA into plant cells, which is ideal for transient protein production in plants [3].

Although a plant-based recombinant protein production system provides distinct advantages over traditional systems, the differences between *N*-glycosylation of proteins produced in plants and humans could limit the use of plant systems for the production of glycoprotein-based pharmaceuticals. In higher eukaryotes, the initial steps of *N*-glycosylation processing are well conserved between plants and human, resulting in oligomannose-type *N*-glycosylation. However, late *N*-glycosylation maturation in the Golgi apparatus is kingdom-specific, and thus results in different *N*-glycosylation on proteins produced in plants compared with human. These plant-specific glycans may lead to potential safety issues such as hypersensitivity or allergy, as plant-specific α(1,3)-fucose and β(1,2)-xylose are known to be important IgE binding determinants of plant allergens [4]. Thus, if these plant-specific glycans are present in an injected pharmaceutical product, the glycoprotein could trigger immunological response, or at least, result in a short circulation half-life. *N*-glycans of proteins produced from mammals are often terminated in β(1,4)-galactose and sialic acid; sialic acid is particularly important as it typically increases the circulation half-life of proteins [5].

There are a number of ways to avoid incorporating plant-specific glycans in the product such as adding a signal sequence (i.e., SEKDEL) at the C-terminus of the target protein to retain it within ER [6], or RNAi-mediated knock-down of α(1,3)-fucose and β(1,2)-xylose [7]. These methods require modification to either the protein sequence or to the expression system, which can potentially affect protein structure and require a long developmental time. As an alternative, the use of small molecule inhibitors of intracellular glycosidases is a highly flexible bioprocessing approach for controlling protein *N*-glycosylation patterns in transient agroinfiltration processes, and it is the approach investigated in this study.

Here, we report an easy and fast way to modify *N*-glycosylation of recombinant proteins produced transiently in *N. benthamiana* through the addition of kifunensine in the *agrobacterium* suspension prior to vacuum agroinfiltration, which avoids modification to protein sequence or expression system while producing recombinant protein with oligomannose-type *N*-glycans that are similar between plant and human. Oligomannose-type *N*-glycan is preferred for the HIV-1 viral vaccine development as a vast majority of broad and potent neutralizing antibody responses during HIV-1 infection target mannose-glycan-dependent epitopes [8]. In addition, monoclonal antibodies with oligomannose *N*-glycans show increased ADCC activity and affinity for FcγRIIIA [9].

Protein *N*-glycosylation starts in the endoplasmic reticulum (ER), where *N*-glycan precursors Glc_3_Man_9_GlcNAc_2_ are first synthesized, followed by the removal of terminal Glc residues, resulting in Man_9_GlcNAc_2_ (Man9) structures. Then, a single α1,2 linked mannose is removed by ER class I α-mannosidase, producing Man_8_GlcNAc_2_ (Man8) structures [10]. The trimming of α1-2 mannose residues continues with the action of Golgi class I α-mannosidases in *cis*-Golgi to give Man5 structures [10]. Kifunensine is a highly selective inhibitor of class I α-mannosidases in both plants and animals [11], and it has been used in cell cultures to produce recombinant proteins with oligomannose-type *N*-glycans [9,12,13,14].

Although the general effects of kifunensine and other alkaloid-like processing glycosidases inhibitors are well understood, for the most part this information comes from cell culture system studies. Meanwhile, the study of kifunensine on whole-plant transient protein expression through agroinfiltration is new. There are only two published papers on whole-plant kifunensine treatment, where kifunensine was supplied hydroponically throughout the whole incubation period [15,16], which requires larger quantities of kifunensine, a more expensive hydroponic system and constant monitoring especially at large scale as compared to our method. In addition, it was also shown that hydroponic kifunensine treatment resulted in dramatic decrease of protein expression level [16] which was not observed with our method.

In this study, Fc-fused capillary morphogenesis gene-2 (CMG2-Fc), an anthrax decoy protein, served as a model protein, which contains single *N*-glycosylation site within its Fc domain (Asn297 of IgG1). CMG2-Fc is a potent anthrax decoy protein as shown previously [17], where the CMG2 domain binds to anthrax protective antigen and prevents the anthrax toxin from entering the cell. Meanwhile, the presence of Fc domain significantly increases the serum half-life, which prolongs therapeutic activity owing the slower renal clearance for larger sized molecules and interaction with the salvage neonatal Fc-receptor [18]. CMG2-Fc thus can be used as potent anthrax therapeutic and prophylactic without frequent redosing. The expression levels of CMG2-Fc produced transiently in wild type *N. benthamiana* under kifunensine treated and untreated conditions were measured with a sandwich ELISA, and protein *N*-glycosylation profiles were evaluated with mass spectrometry for kifunensine treated and untreated conditions.

The findings in this study can be applied for *N*-glycosylation modification of other plant recombinant proteins when oligomannose-type *N*-glycans lacking core fucose are preferred, without the need to modify protein sequence and/or subcellular targeting.

## 2. Results

### 2.1. Expression of CMG2-Fc in Plant Leaves

The CMG2-Fc expression levels in crude leaf extract were quantified through a sandwich ELISA to confirm the expression of CMG2-Fc, and to evaluate the effect of kifunensine on protein expression. The ELISA relies on binding of CMG2-Fc through the Fc region to protein A coated on a 96-well plate. A secondary anti-Fc polyclonal antibody linked to a horseradish peroxidase (HRP) enzyme binds to the CMG2-Fc allowing colorimetric detection. The potential interference of plant host cell proteins and nonspecific binding were determined to be negligible.

**Figure 1:**
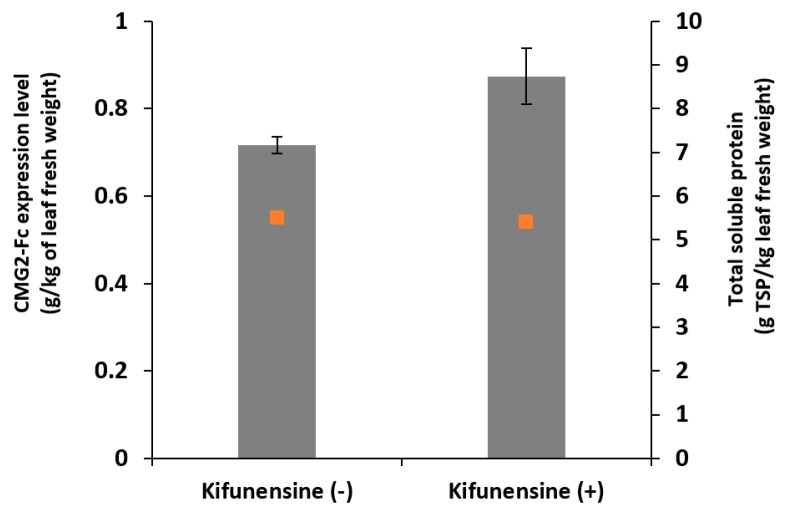
Expression levels of CMG2-Fc at day 6 post-infiltration as measured by ELISA (grey bars) and total soluble protein (TSP) in crude plant extract (orange dots). Kifunensine (−) stands for the control group without addition of kifunensine in the agrobacterium suspension; kifunensine (+) stands for the experimental group with 5.4 µM of kifunensine in the agrobacterium suspension. The CMG2-Fc levels are presented on the leaf fresh weight (FW) basis. Error bars represent standard deviations of duplicated ELISA measurements and duplicated Bradford measurements.

Twenty wild-type 5–6-week old *N. benthamiana* plants were divided equally into experimental and control groups, agro-infiltrated and incubated for 6 days, then whole leaves were extracted under identical conditions to determine protein expression. Kifunensine at a concentration of 5.4 µM was included in the *agrobacterium* suspension in the Kifunensine (+) group. This kifunensine concentration was chosen as a starting point by taking the average of concentrations used in a previous CHO cell culture study [9], as no reference concentration is available for vacuum infiltration of kifunensine. The average mass of CMG2-Fc per kg leaf fresh weight (FW) was 717 and 874 mg/kg leaf FW for the Kifunensine (−) and Kifunensine (+) samples, respectively (Figure 1). The control group expression level was consistent with previous results [19]. These data suggest that the addition of kifunensine in the agro-infiltration process was not detrimental to transient protein production, and in this case, it resulted in a 22% increase in CMG2-Fc yield. This allows the use of kifunensine for modification of glycosylation profiles without compromising protein expression. Total soluble protein content of whole leaf extract of Kifunensine (−) and Kifunensine (+) groups were similar as shown in Figure 1, which indicates that kifunensine does not have significant impact on plant protein synthesis in general.

### 2.2. Effects of Kifunensine on the N-Glycan Profile of CMG2-Fc Produced in Nicotiana Benthamiana

The *N*-glycosylation profiles of CMG2-Fc transiently expressed in *N. benthamiana* were evaluated by LC-MS/MS analysis. Samples were prepared in two ways: whole leaf extract and AWF recovery, so that glycoforms of both intracellular and extracellular CMG2-Fc can be analyzed. Whole leaf extract sample was purified with Protein A chromatography prior to *N*-glycan analysis. The *N*-glycan pattern of kifunensine-treated and untreated samples was compared to evaluate the inhibition efficacy of kifunensine when added to the *agrobacterium* suspension prior to vacuum infiltration.

**Figure 2:**
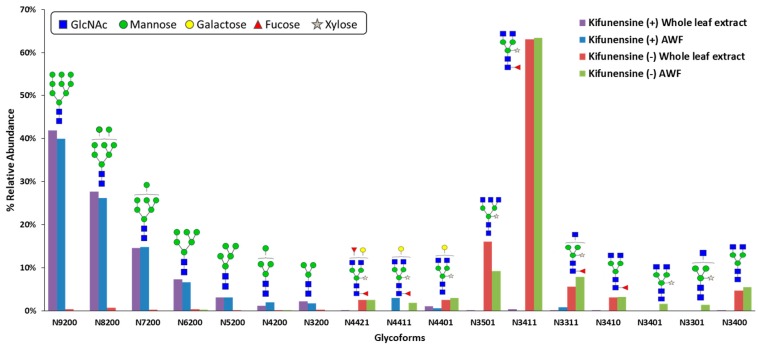
*N*-Glycan structures analysis of CMG2-Fc with/without Kifunensine treatment from whole-leaf extract and AWF samples. The relative abundance of glycoforms is represented in this bar chart. The annotations on *x*-axis represent numbers of N-linked glycan molecules: mannose and galactose/GlcNAc/Fucose/Xylose residues, respectively. Red and green bars: without kifunensine, resulted in addition of complex-type *N*-glycans. Purple and blue bars: vacuum infiltrated with kifunensine at 5.4 µM, resulted in predominantly oligomannose-type *N*-glycans, Man_3–9_GlcNAc_2_.

The *N*-glycosylation profile of CMG2-Fc produced without kifunensine exhibited primarily complex-type *N*-glycans (97.7% for whole leaf extract and 99.7% for AWF), with the most abundant *N*-glycan structure of GlcNAc_2_(Xyl_1_)Man_3_(Fuc)GlcNAc_2_. There was no significant difference in CMG2-Fc *N*-glycan profiles between untreated whole leaf extract and AWF samples as shown in Figure 2. These observations indicated that CMG2-Fc produced transiently in *N. benthamiana* was fully glycosylated, and consequently, was secreted to apoplast as expected. As for kifunensine-treated plants, the abundances of complex-type *N*-glycans of CMG2-Fc were 1.8% and 4.4% for whole leaf extract and AWF, respectively, resulting in an almost complete shift of *N*-glycan profile from complex-type to oligomannose-type under kifunensine treatment. The most dominant *N*-glycan was Man_9_GlcNAc_2_ for both whole leaf extract (41.9%) and AWF (40.0%), which suggested that kifunensine inhibition activity toward α-mannosidases I was sustained throughout the protein expression period.

## 3. Discussion

In this study, the influences of one-time kifunensine vacuum infiltration on the expression level, *N*-glycan profile of a recombinant protein, namely CMG2-Fc, produced transiently in *N. benthamiana* plants were evaluated in both whole-leaf extract and AWF.

We found that kifunensine had a positive impact on protein production when supplied in the agroinfiltration solution; specifically, we observed a 22% increase of protein expression with kifunensine treatment condition, presumably owing to its suppressing effects on ER-associated degradation pathway [12]. This finding is consistent with previous observations in multiple mammalian cell culture systems [9,12,13], and there is no reason to suspect that this will not be the case for other eukaryotic systems, including plant systems. Plants were monitored visually throughout the incubation period, and there were no significant phenotypical differences between kifunensine-treated and control groups.

In the case of a whole-plant study, Roychowdhury et al. have shown that the yield of recombinant cholera toxin B subunit dropped by 30% and 75% when kifunensine was supplied at 5 µM hydroponically for 3 days and 5 days post agroinfiltration, respectively [16]. In contrast, we observed slight increase in protein yield when kifunensine was infiltrated in leaf tissue instead of being supplied hydroponically. Thus, the lower protein yield they observed may have resulted from continued application in the hydroponic solution, and it is eliminated when kifunensine was supplied directly to leaf tissue through vacuum infiltration. In addition, in the hydroponic study, the target protein was retained in ER, while the model protein in our study and other cell culture studies were targeted for secretion [9,12,13,14]. This difference in protein targeting may also play a role in protein yield changes upon kifunensine treatment.

Since kifunensine targets class I α-mannosidases and *N*-glycosylation is an enzyme-directed site-specific process, it was expected that the kifunensine treated plants will produce CMG2-Fc with predominantly oligomannose-type *N*-glycans. It was not obvious, however, that one-time vacuum infiltration provided enough kifunensine and it remained active for the whole protein production period, resulting in a complete shift of *N*-glycan profile from plant-specific complex-type to oligomannose-type. This *N*-glycan profile shift suggested that either kifunensine remains active throughout the entire incubation/production period, or that the plants only express protein for a brief period during which the kifunensine is active. Previous expression kinetics of CMG2-Fc in *N. benthamiana* suggested that protein accumulated in leaf increased from day 1 to day 6 [19], supporting the first explanation. It is likely that kifunensine will remain stable even longer if longer incubation period is desired to maximize protein yield [14].

Kifunensine is known to inhibit enzymatic activity of class I α-mannosidases, and thus should stop mannose trimming in the first place to yield single Man9 *N*-glycan structures. However, we observed multiple oligomannose-type *N*-glycans with mannose residues ranging from 3 to 9, although the most abundant structure was Man9. This observation is consistent with cell culture kifunensine studies [9,13,14], where multiple oligomannose-type *N*-glycans were detected under kifunensine treatment. This could potentially due to the difference in inhibition efficacy of kifunensine towards class I α-mannosidases isoforms, which results in an incomplete inhibition of mannose trimming from Man9 structure. Also taking enzyme kinetics into consideration, depending on the ER concentrations of Man9 glycoprotein substrate, class I α-mannosidases, and kifunensine, enzymatic mannose trimming from Man9 could take place even if the amount of active ER α-mannosidase I is low. Although mannose trimming was not completely inhibited at Man9 structure, this method still showed the ability to significantly modify glycosylation using a simple bioprocessing approach. It is likely that Man9 abundance can be further increased if treated with higher concentration of kifunensine, but it is not necessary if the goal is to eliminate the production of plant-specific complex *N*-glycans.

Although in this case, CMG2-Fc can be purified easily from whole-leaf extracts through a one-step purification with Protein A chromatography, in many cases, multiple steps of chromatography are required to purify a target protein from a large pool of host native proteins when a highly selective affinity tag is not present. This could result in low protein yield and difficulties to achieve high purity, which is typically required for therapeutic recombinant protein products. Targeting proteins to the apoplast allows the collection of target protein in AWF, which contains much lower levels of plant native proteins than whole-leaf extract since only secreted proteins are collected, thus lowers the downstream process complexity. In this case, CMG2-Fc purity and concentration increased by 3.9-folds and 4.4-folds, respectively, when collected in AWF versus in whole-leaf extract. A similar trend was observed in kifunensine-treated samples, which confirms that kifunensine does not affect protein secretion, allowing secretion of CMG2-Fc with oligomannose-type glycoforms. The increase in purity and concentration was consistent with a previous study on harvesting a target protein from plant AWF [20]. Hence, AWF collection is a feasible method for recombinant protein harvesting, which avoids contamination with intracellular host cell proteins, and is particularly valuable when target protein is hard to purify. Together with kifunensine treatment, apoplast-targeted recombinant protein without any plant-specific glycoforms can be transiently produced in *N. benthamiana*, and likely in other plants as well. Products can be collected at high concentration and purity from AWF, containing predominantly oligomannose-type *N*-glycans.

Further studies should focus on determining how long the inhibition effect of kifunensine lasts after the one-time vacuum infiltration by monitoring the protein glycoform profile at multiple time points after vacuum infiltration, and the threshold concentration of kifunensine that results in a complete *N*-glycan shift from plant complex-type to oligomannose-type for other glycoproteins, particularly those with more N-linked glycosylation sites. In addition, the protein expression kinetics should be compared between kifunensine-treated and untreated groups to maximize target protein yield. Depending on the desired glycoform, this method can also be applied to other *N*-glycan processing inhibitors such as castanospermine [21], deoxynojirimycin [22], and swainsonine [23].

## 4. Materials and Methods

### 4.1. Gene Construct Design for Expression of CMG2-Fc

The gene encoding the extracellular domain of CMG2 (amino acids 34-220, Genbank AY233452), followed by two serines, the hinge of human IgG2 and Fc region of human IgG1 was codon-optimized for expression in *N. benthamiana*. The full construct is comprised of the CaMV 35S promoter, Ω leader sequence, Ramy3D secretion signal peptide (targeting the CMG2-Fc protein for secretion) followed by gene encoding CMG2-Fc and octopine synthase terminator. The resulting binary expression vector (pDP16.0707.07) was transfected into *Agrobacterium tumefaciens* EHA105, with the helper plasmid (pCH32) via electroporation.

### 4.2. Transient Expression of CMG2-Fc in N. Benthamiana

The transient expression of CMG2-Fc is achieved by whole-plant vacuum infiltration of *N. benthamiana* with *Agrobacterium tumefaciens* EHA 105 containing binary vector for CMG2-Fc expression and *Agrobacterium tumefaciens* containing the pBIN binary vector for expression of the RNA silencing suppressor P19 from *Tomato bushy stunt virus* [24]. Each *A. tumefaciens* strain was cultured separately in 20 mL of Luria-Bertani (LB) media with selection antibiotics for 18 h in the dark at 28 °C, on an orbital shaker at 250 rpm. Then, 8 mL of each culture is transferred to 250 mL LB media and incubated for 18 h in the dark at 28 °C at 250 rpm shaking to further amplify the cell population. The *A. tumefaciens* cells were collected by centrifugation at 1800× *g* for 30 min at 15 °C, and the cell density was quantified through absorbance measurement at 600 nm. Both *A. tumefaciens* strains were resuspended into infiltration buffer (10 mM MES buffer at pH 5.6, 10 mM MgCl_2_ and 150 µM acetosyringone, and 0.02% *v*/*v* Silwet-L-77) with a final cell density of 0.4 for each strain (OD_600_). Six-week old *N. benthamiana* plants were turned upside down and submerged into the agrobacteria suspension, followed with vacuum infiltration for 1 min after vacuum pressure reached 20 inches Hg. Infiltrated plants were then incubated in a growth chamber at 20 °C and 90% humidity for 6 days. All leaves were cut from the petioles were used for AWF collection or stored at −80 °C for whole leaf protein extraction.

### 4.3. Kifunensine Treatment

Twenty *N. benthamiana* plants were separated equally into control and experimental groups. For the experimental group, kifunensine (Caymon Chemical, Ann Arbor, MI, USA) was added to *Agrobacterium* suspension at concentration of 5.4 µM, which was infiltrated into plant tissue during the agroinfiltration process. For the control group, plants are vacuum infiltrated with agrobacteria suspension without kifunensine.

### 4.4. Apoplast Wash Fluid Collection

Freshly harvested *N. benthamiana* leaves were vacuum infiltrated with extraction buffer (1X PBS buffer, 1 mM EDTA, 2 mM sodium metabisulfite, and 0.02% *v*/*v* of Silwet-L-77) for 1 min after vacuum pressure reaches 20 inch Hg. Leaves were mounted on 50 mL falcon tubes as described by Madsen, S. et al. [25], then centrifuged at 540× *g* for 10–15 min at 4 °C to collect apoplast wash fluid. 

### 4.5. Whole Leaf Extraction

Leaves stored at −80 °C were ground to fine powder in a pre-chilled containing liquid nitrogen with a pestle. The resulting leaf powder was weighed and mixed with the extraction buffer (1× PBS buffer, 1 mM EDTA and 2 mM sodium metabisulfite) at 1:7 leaf mass (g) to buffer volume (mL) ratio. The extract was placed on a shaker at 4 °C for 1 h to extract soluble proteins into the extraction buffer. The crude extract was centrifuged at 1800× *g*, at 4 °C for 1 h, and then filtered through a 0.22 µm filter.

### 4.6. CMG2-Fc Purification

CMG2-Fc was purified from whole leaf extract using Protein A chromatography (GE Healthcare). Whole leaf extract after 0.22 µm filtration was loaded to the column, followed with column wash with 15 column volume of 1× PBS buffer before elution. CMG2-Fc was eluted with 0.1 M glycine-HCl buffer at pH 3 with fraction volume of 1 mL. CMG2-Fc fractions were immediately neutralized with 1 M Tris buffer at pH 8.

### 4.7. Bradford Quantification of Total Soluble Protein in Plant Extract

The total soluble protein concentration in crude was measured with Bradford essay using the Quick Start Bradford 1× protein dye reagent (Bio-Rad, Hercules, CA, USA) at 5× dilution. A standard curve was generated from bovine serum albumin (BSA) at concentrations from 0–0.5 mg/mL with increments of 0.05 mg/mL. The BSA standards, samples, and diluted samples were loaded into a 96-well microplate at a volume of 10 µL. The Bradford dye (190 µL) was then added to each well, allowing color to develop for 2 min prior to reading the absorbance measurement at 565 nm with a Spectramax 34C microplate reader (Molecular Devices, San Jose, CA, USA).

### 4.8. ELISA Quantification of CMG2-Fc Production Level in Crude Extracts

Microplate wells were coated with unlabeled Protein A (Southern Biotech, Birmingham, AL, USA) at a concentration of 0.05 mg/mL in phosphate-buffered saline (PBS) at 37 °C for 1 h, and then blocked with 5% nonfat dry milk in PBS buffer. Crude plant extract and purified CMG2-Fc standards (at concentration ranging from 0–0.5 µg/ML, 50 µL each) were added to wells and incubated at 37 °C for 1 h. CMG2-Fc bound to Protein A on the plate was detected by adding 50 µL of horseradish peroxidase (HRP)-labeled goat anti-human IgG (Southern Biotech, 0.5 mg/mL) at concentration of 0.4 µg/mL and incubated for 1 h at 37 °C. Between each of these steps, microplates were washed with PBS with 0.05% *v*/*v* of Tween-20. Finally, the protein concentration was quantified by adding 100 µL of TMB substrate (Promega, Madison, WI, USA). Plates were incubated at room temperature for 10 min, followed by addition of 100 µL 1N HCl to stop the reaction. The TMB substrate reacts with HRP, allowing colorimetric detection of CMG2-Fc levels. The absorbance was measured at 450 nm with a Spectramax 34C microplate reader (Molecular Devices).

### 4.9. Protein Glycoform Analysis with Mass Spectrometry

Site-specific N-linked glycosylation analysis including measurement of the glycopeptide relative abundance of the protein was analyzed with mass spectrometry. Samples were first reduced with 2 µL of 550 mM dithiothreitol (DTT) at 65 °C for 50 min to break the disulfide bond between cysteine amino acids. Then 4 µL of 450 mM iodoacetamide (IAA) was added as an alkylation reagent for 25 min. Samples were placed in the dark environment to prevent the loss of IAA efficacy. After denaturing, proteins were digested with 1 µg of trypsin for 18 h in a 37 °C water bath. The digestion process was stopped by placing samples at −20 °C for around one hour. An Agilent 1290 infinity ultra-high-pressure liquid chromatography (UHPLC) system coupled to an Agilent 6495 triple quadrupole (QQQ) mass spectrometer was used for *N*-glycosylation analysis. For sample separation, the analysis column used on the UHPLC system was an Agilent Eclipse plus C18 column (RRHD 1.8 µm, 2.1 × 150 mm). To protect the analysis column, an Agilent Eclipse plus C18 trap column (RRHD 1.8 µm, 2.1 × 5 mm) was used to trap samples first. The mass spectrometer was operated using the dynamic multiple reaction monitoring (MRM) mode which is a targeted tandem MS mode and Agilent MassHunter Quantitative Analysis B.05.02 software was used for data analysis. In the MRM method, glycopeptides were quantified with the glycopeptide mass used as the precursor ion and the common oxonium fragments with *m*/*z* 204.08 and 366.14 used as product ion. The concentration of glycopeptide in ion counts was normalized to the total ion counts of glycopeptides in the sample for the relative abundance calculation.

## Abbreviations

Glc	Glucose
GlcNAc	*N*-Acetylglucosamine
Man	Mannose
Xyl	Xylose
Fuc	Fucose
TMB	3,3’,5,5’-Tetramethylbenzidine
MES	2-(*N*-morpholino)ethanesulfonic acid
